# The effect of amantadine on an ion channel protein from Chikungunya virus

**DOI:** 10.1371/journal.pntd.0007548

**Published:** 2019-07-24

**Authors:** Debajit Dey, Shumaila Iqbal Siddiqui, Prabhudutta Mamidi, Sukanya Ghosh, Chandra Shekhar Kumar, Soma Chattopadhyay, Subhendu Ghosh, Manidipa Banerjee

**Affiliations:** 1 Kusuma School of Biological Sciences, Indian Institute of Technology Delhi, India; 2 Department of Biophysics, University of Delhi (South Campus), Delhi, India; 3 Institute of Life Sciences, Bhubaneswar, India; Faculty of Science, Ain Shams University (ASU), EGYPT

## Abstract

Viroporins like influenza A virus M2, hepatitis C virus p7, HIV-1 Vpu and picornavirus 2B associate with host membranes, and create hydrophilic corridors, which are critical for viral entry, replication and egress. The 6K proteins from alphaviruses are conjectured to be viroporins, essential during egress of progeny viruses from host membranes, although the analogue in Chikungunya Virus (CHIKV) remains relatively uncharacterized. Using a combination of electrophysiology, confocal and electron microscopy, and molecular dynamics simulations we show for the first time that CHIKV 6K is an ion channel forming protein that primarily associates with endoplasmic reticulum (ER) membranes. The ion channel activity of 6K can be inhibited by amantadine, an antiviral developed against the M2 protein of Influenza A virus; and CHIKV infection of cultured cells can be effectively inhibited in presence of this drug. Our study provides crucial mechanistic insights into the functionality of 6K during CHIKV-host interaction and suggests that 6K is a potential therapeutic drug target, with amantadine and its derivatives being strong candidates for further development.

## Author summary

Chikungunya fever is a severe crippling illness caused by the arthropod-borne virus CHIKV. Originally from the African subcontinent, the virus has now spread worldwide and is responsible for substantial morbidity and economic loss. The existing treatment against CHIKV is primarily symptomatic, and it is imperative that specific therapeutics be devised. The present study provides detailed insight into the functionality of 6K, an ion channel forming protein of CHIKV. Amantadine, a known antiviral against influenza virus, also inhibits CHIKV replication in cell culture and drastically alters the morphology of virus particles. This work highlights striking parallels among functionalities of virus-encoded membrane-interacting proteins, which may be exploited for developing broad-spectrum antivirals.

## Introduction

Chikungunya fever is a severe and debilitating illness caused by the mosquito-borne arbovirus, Chikungunya Virus (CHIKV) [1–4]. Infections are generally non-fatal, but this virus has been much in the limelight lately, due to its rapid spread and outbreaks worldwide [2–4]. Although, India is endemic for CHIKV; outbreaks do occur, the latest one reported in New Delhi in 2016 [4]. Currently, there are no specific therapeutics against CHIKV infections, the treatment being primarily symptomatic [1].

CHIKV, like other alphaviruses, is an enveloped RNA virus with particle diameter ranging between 65–70 nm [5]. The viral genome is organized into structural and non-structural protein-encoding regions [5]. The structural protein cassette is composed of glycoproteins (E1-E2-E3), capsid protein (C), and 6K, which was recently shown to have a transframe variant (TF) [6, 7]. 240 copies each of glycoproteins E1 and E2; as well as the capsid protein, are arranged in accordance with T = 4 symmetry [8]. Although the roles of capsid and envelope proteins in the life cycle of CHIKV are fairly well studied [9–14], reports pertaining to the direct functional characterization of 6K are rare, making it the least understood amongst all CHIKV structural proteins. A recent report suggests that 6K is a prime target for mounting CTL- mediated immune response in the host, indicating its significance as a therapeutic target [15]. One contributing factor for the lack of direct biochemical characterization of CHIKV 6K is its extreme intrinsic hydrophobicity, as well as cytotoxicity [7, 16], which makes the production of sufficient quantity of functionally active, recombinant protein fairly challenging. In fact, the majority of the studies on 6K from other alphaviruses—demonstrating the biochemical properties and role in viral life cycle—have been carried out by analysis of mutated virus [17–23] or by RNA expression at the cellular level [7, 24], with only a few studies characterizing recombinant 6K [16, 25]. Further, the molecular details of membrane association by 6K and the exact role of this activity in promoting virus budding remains unknown.

Recently, a transframe (TF) variant of 6K was identified, which is generated from the 6K gene as a result of a (-1) ribosomal frameshift. TF has the same N-terminal domain as 6K, but a different C-terminus [6, 7]. 6K and TF are produced during infections by most alphaviruses, and both are thought to be essential for virus budding, although only TF appears to be packaged within virions while 6K is probably retained at the membranes of infected cells [6, 7]. Additionally, studies with SINV have highlighted the role of palmitoylation for the localization of TF to the plasma membrane [26].

The membrane permeabilization activity of alphavirus 6K places it in the category of viroporins. Nucleation of aqueous passageways by viroporins allows movement of ions and small molecules, which in turn, facilitates virus entry, replication, and egress. Some well-characterized members of the viroporin family include M2 of influenza A virus, Vpu of HIV, p7 of HCV, and 2B of picornaviruses [27]. The role of viroporins in sustaining infections, and their significance as targets for drug development, is illustrated by the antiviral activity of amantadine, which targets the ion-channel forming protein M2 and prevents Influenza A infections [28].

Given the recent scenario of frequent outbreaks of Chikungunya fever in different parts of the world [1–4], and the role of 6K in supporting alphavirus infections [17–23], we attempted to functionally characterize CHIKV 6K and assess its potential as a target for drug development. Using a combination of electrophysiology, cryo-electron microscopy, biophysical techniques, and molecular dynamics simulations, we demonstrate that CHIKV 6K interacts with membranes in multifaceted ways, leading to permeability as well as vesicle fusion. We show that CHIKV 6K exists in oligomeric forms, and forms ion channels in membranes. In addition, we demonstrate that virus-like particles (VLPs) of CHIKV show a marked deviation from their usual morphology when treated with amantadine; and that the inhibitory activity of amantadine extends to CHIKV replication in cell culture. Thus, it can be strongly emphasized that 6K is critical in CHIKV biology and is a potential therapeutic target for the treatment of Chikungunya fever.

## Results

### 6K is a highly hydrophobic protein which migrates as a hexamer in size exclusion chromatography

**Fig 1:**
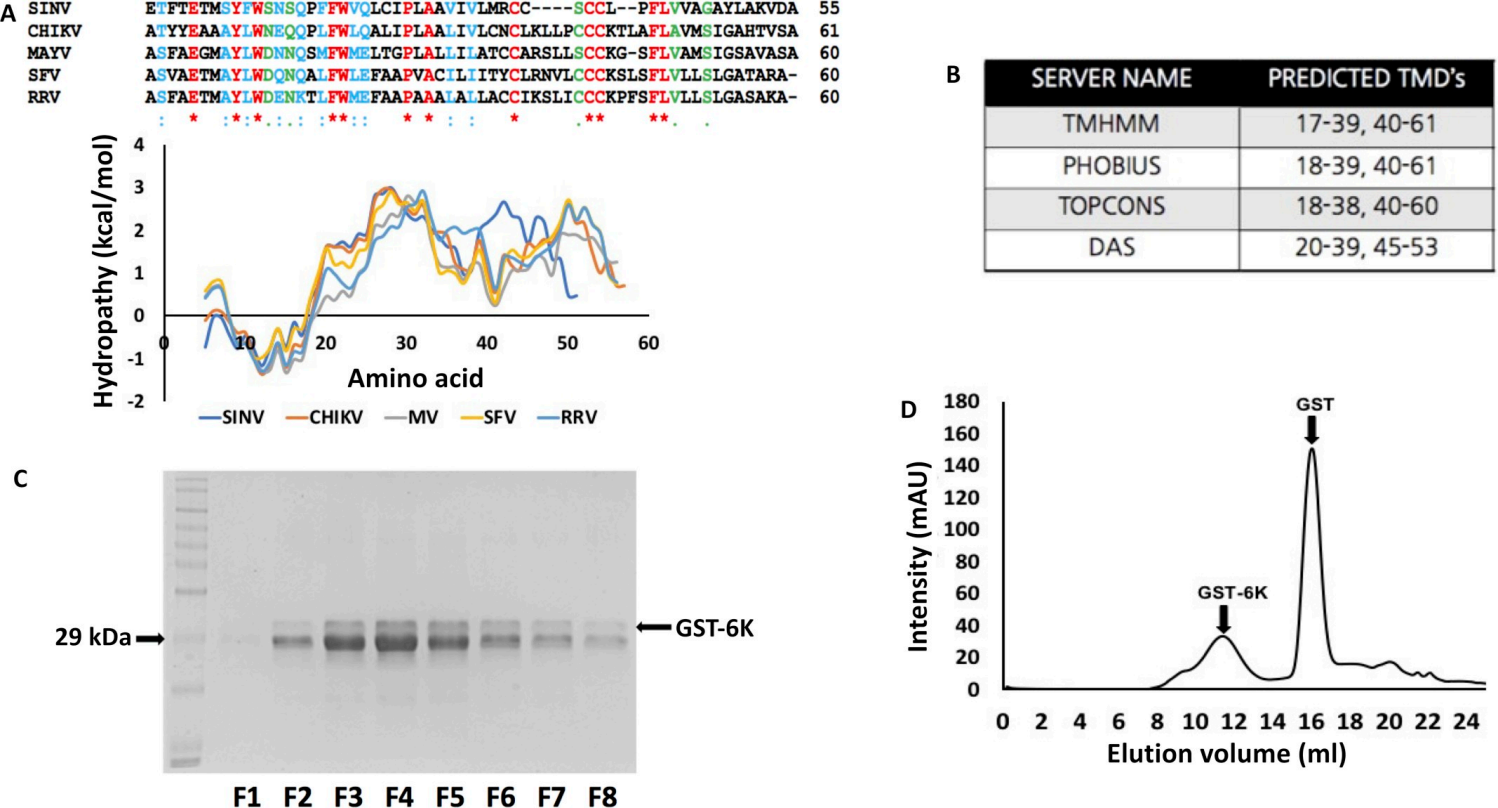
6K sequence analysis and GST-6K purification: (A) A comparative analysis of the hydrophobicity of 6K peptides encoded by different members of the alphavirus family. Kyte-Doolittle hydrophobicity plots and multiple sequence alignment of 6K peptides from different alphaviruses—Chikungunya (CHIKV), Semliki Forest Virus (SFV), Sindbis Virus (SINV), Ross River Virus (RRV) and Mayaro Virus (MV) are shown. Sequence alignment color legend: red-identical residues; blue-highly similar residues and green-less similar residues. (B) Potential transmembrane domains in CHIKV 6K as predicted by different servers as indicated. (C) SDS-PAGE of GST-6K after pulldown with GST beads. Lane 1 corresponds to the molecular weight ladder, while lanes 2 to 9 correspond to protein fractions. (D) Gel filtration profile of GST-6K on Superdex 200 10/300 G/L column.

The 6K proteins of alphaviruses are extremely hydrophobic in nature with more than 50% of the residues having a positive value on the hydrophobicity scale (Fig 1A). A GRAVY (grand average of hydropathy) value of 1.006 also indicates that the protein has an overall hydrophobic nature. This, as well as the presence of transmembrane regions predicted by several servers (Fig 1B), were likely responsible for our inability to generate CHIKV 6K alone, in soluble form, by recombinant expression in bacteria.

A fusion of CHIKV 6K with GST (Glutathione S Transferase) was successfully expressed and purified from *E*. *coli* Rosetta (pLysS cells). We found that IPTG induction and subsequent bacterial growth at a relatively lower temperature of 18°C, is an essential requirement for maximizing expression. GST-6K was initially purified by pulldown with GST beads (Fig 1C), followed by size-exclusion chromatography, which generated two separate peaks corresponding to GST and GST-6K (Fig 1D). The GST-6K peak was fairly broad and the molecular weight corresponding to the primary peak fraction (Fig 1D, indicated with an arrow) was calculated to be 190.98 kDa, which indicated the formation of a hexamer (monomer MW = 33.5 KDa). Since the recombinant protein appeared to have the propensity to break into its constituent parts (Fig 1D), GST-6K was utilized within 24–48 hours post purification for every experiment.

### CHIKV 6K forms ion channels in lipid bilayers

**Fig 2:**
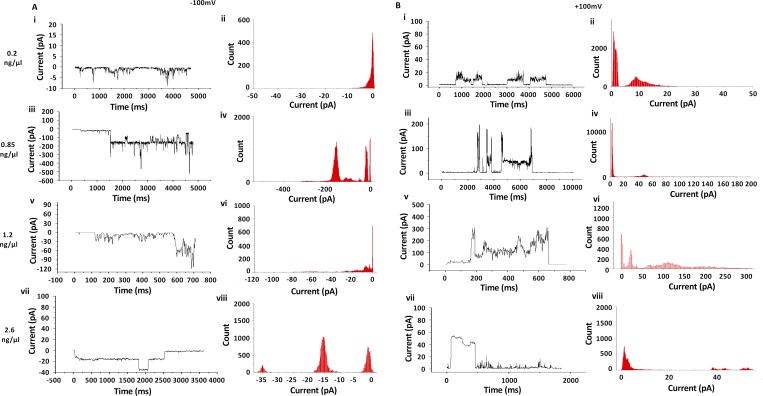
Ion channel activity of GST-6K: (A) and (B) represent ion channel activity of recombinant GST-6K on DPhPC bilayer membrane at applied membrane potentials of **-**100mV and +100 mV respectively. The rows from top to bottom represent increasing concentrations of GST-6K (0.2, 0.85, 1.2 and 2.6 ng/μl) added to the BLM, whereas in each case the current vs time trace and the corresponding histograms are represented side-by-side (For e.g. Fig 2A—i and ii).

The ability of GST-6K to form ion channels *in vitro* was investigated using an electrophysiology setup as described in Materials and Methods. The ion channel activity of GST-6K was measured as a flow of current across an otherwise intact DPhPC membrane (Fig 2A and 2B). Fig 2A and 2B (i, iii, v and vii) represent the current versus time traces of GST-6K incorporated into the lipid bilayer membrane at applied membrane potentials of -100 mV and +100 mV respectively. The corresponding all point histograms are shown in Fig 2A and 2B (ii, iv, vi and viii). Addition of GST-6K to the membrane caused spikes in the current trace, which displayed the typical stepped nature of the opening and closing of an ion channel. This showed that GST-6K successfully incorporated within the bilayer and formed ion-conducting, stable channels, which were functional under the aforementioned membrane potentials, and opened at both negative and positive voltages.

Altogether, observations were made at four different protein concentrations of 0.2, 0.85, 1.2 and 2.6 ng/μl. The lipid (DPhPC) concentration remained fixed in all cases, as the formation of bilayer membrane in the BLM cup demands an exact amount of lipid. Increasing concentration of GST-6K coincided with the appearance of multiple open states with higher currents, at both positive and negative membrane potentials. These observations can be best explained as follows. GST-6K forms pore on the membrane by oligomerization (S1 Fig), however, the number of monomers which oligomerize to form a pore is not fixed. Increase in protein concentration may result in the generation of higher-order oligomers, leading to the formation of bigger pores in the membranes and higher currents (S1 Table). Generation of multiple oligomeric states is well evidenced in pore-forming proteins [29]. Similar experiments with only GST as negative control do not result in the formation of ion channels, as seen by us (S2 Fig) and others [30]. This indicates that the ion-channel forming activity is exclusively due to 6K.

### CHIKV 6K disrupts liposomes mimicking Endoplasmic Reticulum (ER) membrane

**Fig 3:**
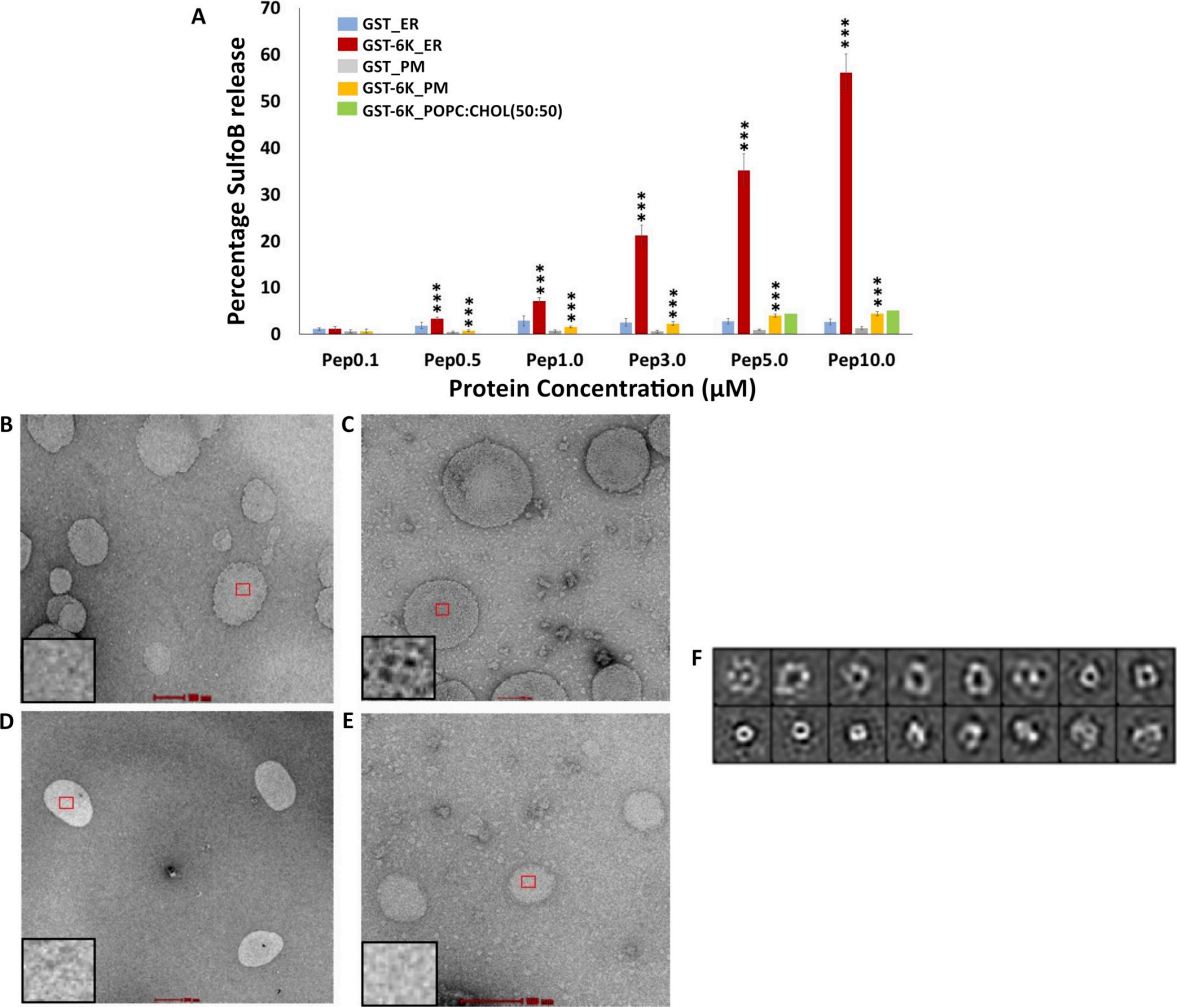
Liposome disruption assays and visualization of GST-6K on organelle mimicking lipid membranes: (A) Plot showing the percentage release of sulforhodamine B from liposomes by GST-6K at concentrations ranging from 0.1–10 μM. Data is represented as mean of triplicate independent samples ± SD (***P < 0.001; ** P < 0.01; * P < 0.05; P >0.05 NS (Non-Significant). (B, C) Negative stain electron micrograph of GST-6K untreated and treated (5 μM) endoplasmic reticulum mimicking liposomes and (D, E) that of plasma membrane mimicking liposomes respectively. Insets show a magnified view of the liposomal surface in each case (B-E). (F) Reference-free 2D classification of negatively stained GST-6K associated with ER liposomes.

Since GST-6K appeared to be capable of forming ion-channels in membranes, we attempted to determine if it preferentially damages target membranes. Liposomes mimicking the lipid composition of ER and plasma membranes encapsulating the fluorescent dye sulforhodamine B, were generated as described [31, 32]. The ability of GST-6K, at a concentration ranging from 0.1 μM-10 μM, to disrupt these liposomes, was tested using standard methods. A distinct increase in disruption of ER mimicking liposomes was observed with concentrations of 0.5 μM (and higher) of GST-6K (Fig 3A). Transmission electron micrographs of GST-6K treated, vs non-treated ER-mimicking liposomes, displayed a clear difference in morphology (Fig 3B and 3C), with the latter image (Fig 3C) showing liposomes with their surfaces decorated with proteinaceous material. Incubation of plasma membrane mimicking liposomes with GST-6K did not cause any significant release of dye (Fig 3A), or alteration of surface features (Fig 3D and 3E), indicating the inability of GST-6K to effectively rupture these membranes.

To further pinpoint the role of specific lipids in GST-6K-mediated membrane damage, similar experiments were conducted with SulfoB-encapsulating liposomes composed of DOPC (Dipalmitoylphosphatidylcholine) and cholesterol in a 1:1 molar ratio. GST-6K, even at relatively higher concentrations of 5 and 10 μM, was incapable of disrupting these liposomes (Fig 3A). These observations, taken together, indicated that CHIKV 6K is probably unable to damage membranes rich in cholesterol. Free, purified GST did not display any significant ability to disrupt membranes, indicating that the GST tag did not influence the effect of CHIKV 6K on membranes in any way (Fig 3A).

### GST-6K appears to form heterogeneous oligomers on ER liposomes

Incubation of ER-mimicking liposomes with GST-6K and subsequent visualization using transmission electron microscopy, revealed that the protein dotted the surface of liposomes (Fig 3C). Manual picking of particles and 2D classification (Fig 3F) indicated that the morphology of the structures formed was not uniform. Thus, it appears that GST-6K may induce the formation of a range of oligomeric structural units on the surface of membranes.

### 6K and E2 localization within cells indicate plasma membrane trafficking

**Fig 4:**
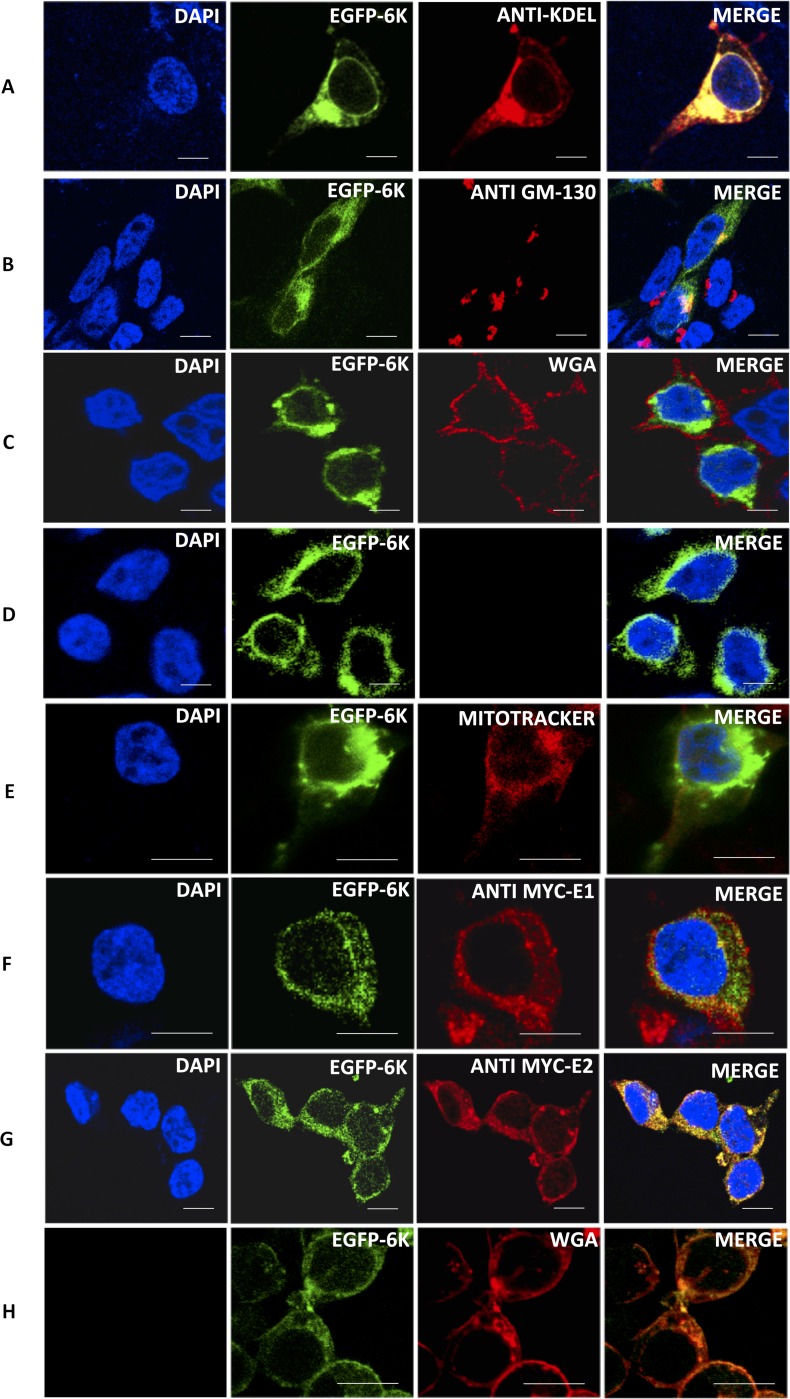
Mammalian cell expression of EGFP-6K shows predominant ER expression: Confocal microscopy images of HEK293T cells expressing EGFP-6K alone or in conjunction with myc-E1 and/or myc-E2 glycoproteins. Panels (A-E) show images of cells expressing EGFP-6K and stained with fluorescent markers corresponding to ER (A, anti-KDEL, red), golgi (B, GM-130, red), plasma membrane (C, red, WGA), nucleus (D, DAPI, blue) and mitochondria (E, mitotracker, red). Panels (F) and (G) contain cells expressing EGFP-6K along with E1(Panel F) and E2 (Panel G) respectively. Panel (H) contain cells co-expressing EGFP-6K and E2 and stained with WGA (red). Scale bars: (A-D, G: 10 μm); (E, F, H: 5 μm).

The inability of CHIKV 6K to cause leakage in plasma membrane specific liposomes was somewhat surprising, as the stipulated role of 6K during virus particle egress implies some association with the plasma membrane [21]. We, therefore, tested the cellular location of 6K tagged with EGFP (Enhanced Green Fluorescent Protein) in mammalian epithelial cells using confocal microscopy. Upon transfection of EGFP-6K into HEK (Human Embryonic Kidney) 293T cells, the GFP fluorescence showed a very high degree of localization with the ER, partial localization with Golgi membranes, but no localization to mitochondria, nucleus or the plasma membrane (Fig 4A–4E). HEK-293T cells were also co-transfected with pCDNA3.1 containing either CHIKV E2 or E1 glycoproteins, along with GFP-6K. The E1 and E2 glycoproteins were expressed in conjunction with an N-terminal myc-tag, which was detected with an anti-rabbit HRP conjugated antibody (Fig 4F and 4G). 6K selectively localized with E2 as opposed to E1 and traversed to the plasma membrane upon simultaneous expression with E2 (Fig 4H). This data, in conjunction with previous instances of E2 trafficking to the plasma membrane by itself [33–35], suggests that the physiological role of 6K in infected cells is quite possibly dependent on E2, which might dictate the trafficking of 6K to the plasma membrane.

### Oligomers of 6K form ion channels in membranes *in silico*

**Fig 5:**
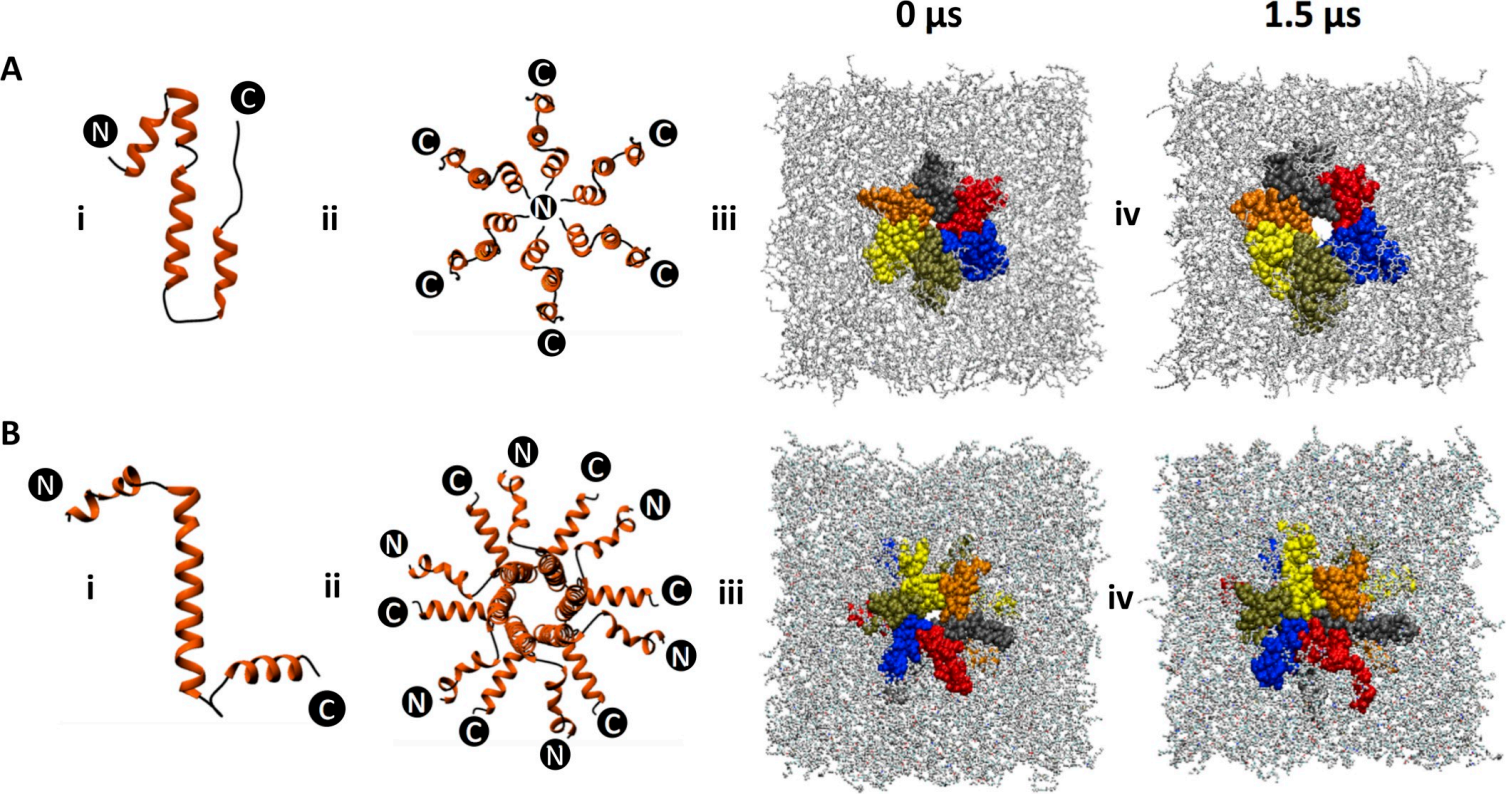
Molecular dynamics simulation of CHIKV 6K oligomers in membrane showing channel activity: Bhageerath-H predicted two possible predicted structures of 6K: 6k_1_ (A-i) and 6k_2_ (B-i) along with their respective oligomeric arrangements (A-ii and B-ii), and snapshots of micro-second scale simulations of 6K embedded within POPC membranes (A-iii, iv and B-iii, iv respectively). Membrane lipids are colored grey (line representation), while 6K monomers are color-coded from peptide1-6 (green-yellow-orange-black-red-blue) in space-filling, van der waal’s representation.

In order to better understand the mode of membrane association and ion channel formation by CHIKV 6K, we carried out microsecond scale (1.5 μs) MD simulation studies. In the absence of an experimentally derived tertiary structure for 6K, two different predicted structures - 6k_1_ and 6k_2_—were considered (Fig 5A-i and 5B-i respectively). All simulations were carried out for a total of 1.5 μs in the presence of 100 mM concentration of NaCl under the influence of an applied electric field.

When a single molecule of 6k_1_ was embedded within a pre-equilibrated POPC (1-pamitoyl-2-oleoyl-sn-glycero-3-phosphocholine) membrane the peptide adopted an overall angular conformation with the central alpha-helical segment (^14^QQPLFWLQALIPLAALIVLCNCLR^37^) attaining an almost 15-degree tilt with respect to its initial conformation. The peptide retained this conformation for the rest of the simulation period.

However, embedding 6 molecules of 6k_1_ in the membrane led to the formation of a small stable channel through which ions were observed to pass through (Fig 5A-iv). Secondary structural analysis of the individual monomers (S3 Fig) showed that 6k_1_ primarily remains helical during simulation time, with the central alpha-helical segment of each monomer spanning through the length of the membrane, thereby providing structural stability to the overall complex. The average size of the channel was found to be ~1 nm, which is sufficient for ions and small molecules like sulforhodamine B to pass through and also corresponds closely to the channel diameter obtained from electrophysiology experiments. We utilized the “Pore Walker” server [36] to detect the residues within the channel and found that the channel lumen was primarily lined by residues Ala^1^, Thr^2^, Tyr^3^, Glu^5^, Ile^24^, Pro^25^, Ala^28^, Leu^32^ and Arg^37^ from each monomer.

Similar simulations studies with the oligomeric form (Fig 5B-ii) of 6k_2_ produced an interesting outcome (Fig 5B-iii, iv). No stable channel for ion passage, unlike that observed for 6k_1_ oligomers, were detected in these cases (Fig 5-iv). A plausible explanation for this discrepancy was provided by the difference in the orientation of the N-terminal helix in 6k_1_ and 6k_2_ conformations (Fig 5A-i and 5B-i respectively). We generated two potential oligomeric arrangements with the best possible spatial placement of 6k_1_ and 6k_2_. The 6k_1_ oligomer contains the N-terminal helices positioned towards the interior of the assembly (Fig 5A-ii), whereas the 6k_2_ oligomer has the N-terminal helices projected outwards (Fig 5B-ii). The hydrophobicity plot for 6K (Fig 1A) clearly shows that, except the small N-terminal stretch of approximately 15–20 residues, the rest of the sequence is considerably hydrophobic. Thus, as the simulation progressed, the inward orientation of N-terminal helices of 6k_1_ probably favored the stabilization of the channel, widening it enough for the passage of small molecules; whereas, for 6K_2_, the cavity collapsed on itself, thus hindering the formation of a stable channel. Our simulation studies provide indications as to how CHIKV 6K forms ion channels within biological membranes and the dynamics of ion conduction and hints towards the critical role of the N-terminal residues of 6K in the formation and stabilization of the ion channel.

### 6K also displays membrane fusogenic properties

**Fig 6:**
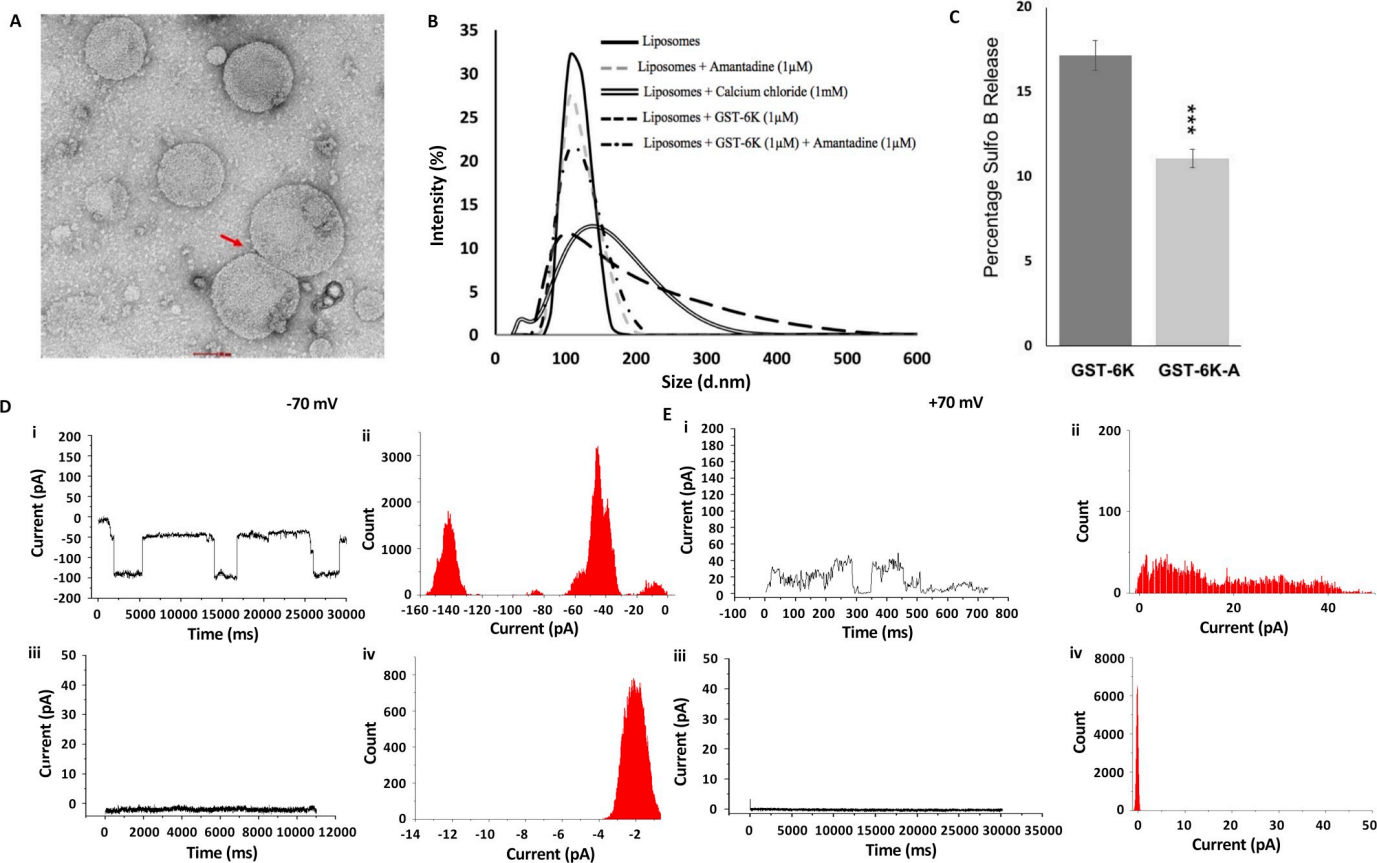
GST-6K ion channel and liposomal fusion activity is inhibited by Amantadine: (A) Negative stained EM image of ER liposomes undergoing fusion upon incubation with 1 μM GST-6K. (B) Dynamic light scattering profile (Intensity vs. size) of liposomes exposed to Ca^2+^ ions, GST-6K, and amantadine. (C) Disruption of ER liposomes by 1μM GST-6K in presence or absence of 1μM amantadine. The plot shows the percentage of disruption of ER liposomes evaluated in terms of the amount of sulforhodamine dye released. Data are represented as mean of triplicate independent samples ± SD (***P < 0.001; P >0.05 NS (Non-Significant), Student’s t-test in comparison with GST-6K only. (D) and (E) Current versus time trace for channel formation by 2.6 ng/μl GST-6K on DPhPC bilayer membrane at negative (-70 mV) and positive (+70 mV) applied membrane potentials. (i**)** and (ii) represent current trace and the corresponding histogram for GST-6K only, while (iii) and (iv) represent the same experiment carried out in presence of 1 μM amantadine in addition to GST-6K.

During the visualization of GST-6K interaction with ER mimicking liposomes by transmission electron microscopy (Fig 3C), the formation of a population of liposomes with larger diameter was noticed. Further studies clearly showed the occurrence of liposomal fusion (Fig 6A), which was further investigated using dynamic light scattering (DLS) (Fig 6B). First, 1 mM calcium chloride was utilized as a positive control, as calcium ions are known to facilitate fusion of artificial vesicles *in vitro* [37]. Addition of calcium chloride resulted in a size-shift of vesicles to larger liposomes (Fig 6B). Upon exposure of ER-mimicking liposomes to 1 μM GST-6K, a distinct overall increase in the diameter of vesicles was noted, indicating the presence of larger sized structures (Fig 6B). This additional, fusogenic ability of CHIKV 6K appears to be similar to that of the M2 channel protein of Influenza A virus [38]. Given these similarities between Influenza A M2 and CHIKV 6K, we attempted to check whether the membrane fusion events orchestrated by CHIKV 6K could be prevented by amantadine, a well-known inhibitor of M2 [38]. The fusion of ER-mimicking liposomes by GST-6K was entirely abrogated by 1μM amantadine (Fig 6B). Addition of the same amount of amantadine also inhibited the release of fluorescent dye from ER mimicking vesicles by GST-6K to a significant proportion (Fig 6C).

### Amantadine inhibits CHIKV 6K *in vitro*

The effect of amantadine on the ion channel formation by GST-6K was checked by electrophysiology experiments described previously at both positive and negative membrane potentials (Fig 6D and 6E), addition of 1 μM amantadine to 2.6 ng/μl GST-6K on the bilayer (BLM) resulted in the formation of only closed states (Fig 6D and 6E, iii-iv), while clear close and open states were detected in the absence of amantadine (Fig 6D and 6E, i-ii). This clearly indicates that amantadine abolishes the ion channel activity of CHIKV 6K. It may be mentioned here that given the concentration of the protein the recordings show that 6K forms multi-channels, which are likely to be due to the formation of different oligomeric states of the protein on the BLM.

### CHIKV VLPs show altered morphology and packaging defects in presence of Amantadine

**Fig 7:**
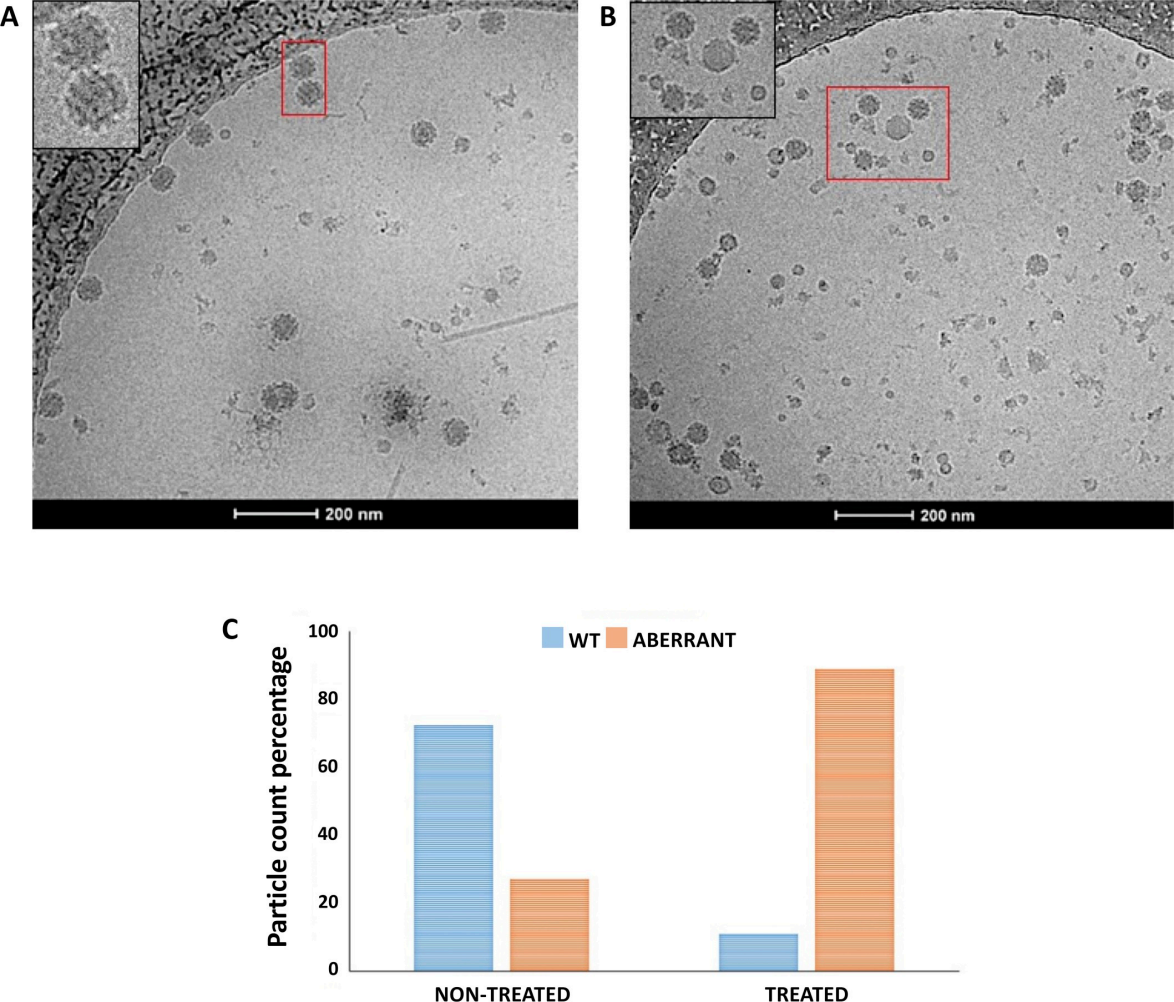
Amantadine treatment alters CHIKV VLP particle morphology: Cryo-electron micrographs of CHIKV VLPs generated from (A) untreated and (B) amantadine treated HEK293T cells transfected with the CHIKV structural cassette. (C) A histogram plot of wild type and aberrant CHIKV particles counted from cryo-micrographs as in (A) and (B) shows the relative abundance of the two species.

To understand whether the ability of amantadine to inhibit the membrane activity of 6K translates to any effect on CHIKV particle assembly, we transfected HEK 293T cells with the cDNA encoding the entire structural protein cassette of CHIKV, followed by treatment of transfected cells with 1μM amantadine. Transfection of the structural protein cassette resulted in the formation of CHIKV VLPs, which were purified and observed using cryo-electron microscopy (Fig 7A). While the majority of particles generated from untreated cells (~74%) displayed the usual size and morphology of wild type CHIKV (Fig 7A), particles generated from amantadine-treated cells were relatively smaller, heterogeneous, and in some cases appeared to lack lipid-associated glycoproteins (Fig 7B). Upon detailed visual examination of ~1000 particles from each population, 89.29% of particles generated from amantadine treated cells exhibited aberrant morphology, in contrast to 24.79% aberrant particles generated from untreated cells (Fig 7C). We postulate that the deviation of CHIKV particles from standard size and morphology is possibly due to the detrimental effect of amantadine on the membrane activity of 6K (Fig 6D and 6E). Taken together, our data highlights the necessity of 6K-mediated membrane interaction for correct virus particle assembly.

**Fig 8:**
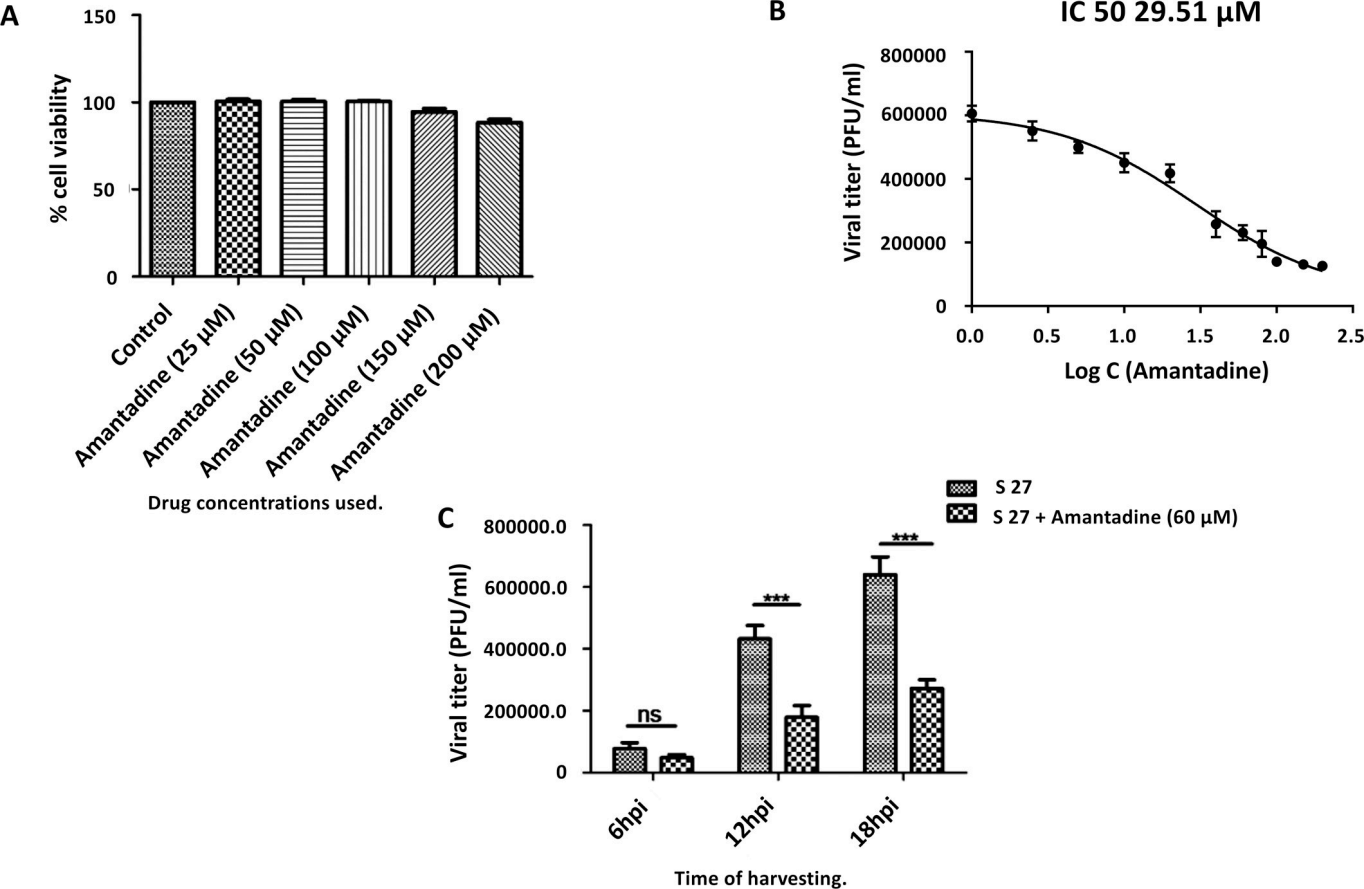
Amantadine inhibits CHIKV infection *in vitro*: (A) Bar diagram showing percent cellular viability in the presence of different concentrations of amantadine as estimated by MTT assay. (B) Representation of CHIKV inhibition curve, where the x-axis depicts the logarithmic value of the concentration of amantadine and y-axis depicts the PFU/ML. Viral titers were determined through plaque assay from cell culture supernatants of CHIKV infected (MOI 0.1) and drug-treated samples harvested at 18 hpi. (n = 3; *p* ≤0.05). (C) Bar graph showing viral titer of cell culture supernatants of CHIKV infected samples (with and without amantadine). For statistical analysis, Two way ANOVA followed by Bonferroni post-test analysis was carried out for determining the level of significance. N = 3; p<0.05).

To check whether the inhibitory activity of amantadine on correct virus assembly extends to the infectivity of virus particles, the ability of CHIKV (strain S 27) to replicate in vero cells was tested in presence and absence of amantadine. First, any cytotoxic effects of amantadine on vero cells was tested with different concentrations of the drug (25–200 μM) for 24 h. MTT assay showed that while ~100% cells were viable upon being treated with 100 μM of amantadine, the cellular viability decreased to 95% upon treatment with 150 μM of the drug, and was further reduced to 88% with 200 μM of the drug (Fig 8A). To check the effect of amantadine on CHIKV replication, a dose kinetics assay was performed (Fig 8B), in which vero cells were either mock infected or infected with CHIKV (MOI 0.1) and treated with different concentrations of the drug (2.5–200 μM). Cell culture supernatants were harvested at 18 hpi and plaque assay, as well as qRT-PCR, were carried out to estimate the virus titer. As observed in Fig 8B, around 58% reduction in virus titer was observed in samples treated with 40 μM concentration, while 77% reduction was observed at 100 μM concentration of amantadine in comparison to control. For further confirmation, qRT-PCR for CHIKV E1 gene was carried out. It was observed that the E1 gene expression was decreased significantly with increased concentration of amantadine as evident from the Ct values shown in Table 1.

**Table 1:** qRT-PCR analysis of the effect of amantadine inhibition on CHIKV (S 27) infection.

Sl. No	Sample	Mean Ct Values
**1**	CHIKV+ DMSO control	16.42
**2**	CHIKV + 10μM (amantadine)	17.45
**3**	CHIKV + 20μM (amantadine)	18.28
**4**	CHIKV + 60μM (amantadine)	19.80
**5**	CHIKV + 100μM (amantadine)	20.54
**6**	CHIKV + 150μM (amantadine)	20.86
**7**	CHIKV + 200μM (amantadine)	20.95

Additionally, for assessing the effect of amantadine on CHIKV propagation at different time points post-infection, a growth kinetics experiment was performed (Fig 8C). Vero cells infected with CHIKV (0.1 MOI) and treated with 60 μM of amantadine, 90 minutes post infection, were harvested at 6, 12 and 18hpi, and the harvested cell culture supernatants were processed through a plaque assay to estimate the viral titer. It was observed that there was ~38% reduction in viral titer at 6hpi and ~60% reduction at 12hpi and 18hpi as shown in Fig 8C. Taken together, the results indicate that amantadine can inhibit viral infection and shows significant anti-CHIKV effect under *in vitro* growth conditions.

## Discussion

Although different virus families have distinct mechanisms for host interaction, however, there exist notable similarities in the processes of entry, uncoating, replication, and egress. Some examples are membrane fusion for cellular entry of enveloped viruses, amphipathic peptide-mediated membrane disruption by non-enveloped viruses, and viroporin-mediated membrane alteration/remodeling for facilitating viral propagation [39–41]. These analogous steps in the virus-host interaction pathway [42, 43] can potentially be targeted for generating broad-spectrum antivirals. This approach may ultimately be more cost-effective than engineering separate therapies against specific viral pathogens.

Viroporins constitute a group of virally encoded membrane-interacting proteins that are crucial for initiating and maintaining successful viral infections [41] and consequently are suitable therapeutic targets. The 6K protein from alphaviruses, due to its ion channel forming ability, and its critical role in facilitating virus budding, has been considered a member of the viroporin family [16, 21, 24]; although the analogue protein from CHIKV has not been characterized functionally. Here, we show for the first time that the 6K protein from CHIKV associates into oligomers and interacts with membranes–two essential features which categorize virally encoded proteins as viroporins. Interestingly, our data show that CHIKV 6K interacts with membranes in multifaceted ways–it can induce ion channel formation, allow passage of small molecules like fluorescent dyes, and also facilitate fusion of vesicles. As this kind of complex membrane association was earlier identified for the M2 protein of Influenza A Virus, also categorized as a viroporin, we attempted to test whether an existing, FDA-approved and marketed M2 ion channel inhibitor–amantadine- can affect the functionality of CHIKV 6K. Our data showed that indeed, amantadine at a concentration of 1μM [45] was able to inhibit ion channel activity, vesicle fusion and membrane permeabilization properties of 6K *in vitro*. We further hypothesized that if 6K is required for correct budding of CHIKV particles from cells, the inhibition of 6K functionality by amantadine would lead to the production of virus particles with a significant degree of aberrance in morphology, similar to the phenotype earlier observed upon deletion of 6K in other alphaviruses [20, 21]. We found that indeed, amantadine at a concentration of 1μM was sufficient to cause structural aberrations in a majority of CHIKV virus-like particles budding out of cells. This effect of amantadine on the activity of CHIKV 6K is surprising, given the lack of primary sequence similarity between 6K and M2 [44]. However, there could be similarities in the tertiary or quaternary folds of M2 and 6K, which might allow amantadine binding and is worthy of further investigation through structural studies.

Given the effect of amantadine on particle morphology, we tested the possibility of utilizing amantadine as an inhibitor of CHIKV infection. Vero cells infected with CHIKV virions (strain S 27), showed a significant decrease in viral titer upon exposure to 5 μM or more amantadine (Fig 7B). This concentration was higher than that required for alteration of particle morphology, which indicates that during infection, there could be other compensatory factors resulting in the generation of infectious particles, or that the morphologically altered particles do retain some infectivity. The IC_50_ value of amantadine was calculated to be 29.51 μM (Fig 7B), which is comparable to that observed for other antiviral drugs [46]. Additionally, the qRT-PCR assay also confirmed that the expression of viral RNA progressively reduced with increase in amantadine concentration. Moreover, through a growth curve analysis, it was observed that the addition of amantadine inhibited CHIKV infection remarkably at later times *in vitro*. This strongly implies that 6K is indeed a valuable target for the development of pan-alphaviral inhibitors, using amantadine and its derivatives as the starting point.

Our work also highlights interesting features of 6K localization and functionality, which are essential for understanding the mechanism of CHIKV assembly. Our liposome assays show that CHIKV 6K preferentially disrupts vesicles that mimic the lipid composition of the endoplasmic reticulum (~ 60% disruption) as opposed to those that mimic the lipid profile of plasma membrane (~10% disruption). Likewise, recombinant expression of 6K in mammalian cells results in the protein being localized primarily in ER, highlighting a preference shown by the protein for the ER membrane. Indeed, it appears that enhanced cholesterol composition, as present in the plasma membrane, is a deterrent for 6K; however, existing literature conjectures that 6K forms ion channels in the plasma membrane [16]. In order to rationalize these contradictory phenomena, we hypothesized that 6K can probably traffic to the plasma membrane, only if it has a binding partner. Specifically, previous studies showing that the E2 glycoprotein of other alphaviruses interact with 6K, that any detrimental alteration in 6K hinders effective glycoprotein processing [17, 18, 19, 21], and that E2 is also capable of traversing to the plasma membrane on its own, led us to conjecture that E2 might play a role in conveying 6K to the plasma membrane. To verify this hypothesis, we carried out confocal microscopy studies of EGFP tagged 6K, in presence of E1 and/or E2 glycoproteins; and found that CHIKV 6K strongly colocalizes with E2 as opposed to E1, and that its primary location is altered to the plasma membrane upon co-expression with E2. It is possible that the functionality of 6K in altering ionic homeostasis at the plasma membrane is contingent upon its alliance with E2 during virus budding, and that 6K in presence of E2 utilizes the trans-golgi network from ER to reach the plasma membrane.

Our data shows that CHIKV 6K has the propensity to form oligomers and can integrate with planar lipid membranes to produce ion channels. However, how these oligomeric associations are stabilized can only be answered from high resolution experimental structural data. All-atom molecular dynamics simulation studies indicate that the diameter of channels formed by 6K oligomers is in the range of 1.0–1.10 nm, which is sufficient for the passage of small ions and fluorescent dyes. A 2D classification of GST-6K particles dotting the surface of ER liposomes also indicated heterogeneity in the structure of particles formed on these liposome surfaces. A close analysis of amino acids forming the lumen of the channel identified a sizeable proportion of residues from the hydrophilic N-terminal region of individual monomers. The rest of the polypeptide, which is highly hydrophobic, appeared to provide the necessary structural anchorage required to prevent the collapse of the channel. The validity of this arrangement for channel formation is highlighted by attempted simulations with an oligomeric form of 6K, where the N-terminal regions of individual monomers extended outwards from the channel. In this particular arrangement, the central helical transmembrane segments collapsed on one another, disrupting the formation of the central channel.

Recent developments in the field have indicated that the transframe or TF variant of 6K may also be involved in membrane penetration and may be packaged in virions [7]; while the fate of 6K is to remain associated with cellular membranes. Since the sequence for ribosomal frameshift is inherent in the 6K cDNA, we expect that a minor proportion of recombinant protein produced by us will also have an altered C-terminus. However, this alteration will consist of incorporation of an expression-vector derived stretch of 7 residues, which is entirely different from the actual C-terminal region of TF derived from the CHIKV genome. This minor population of recombinantly generated variant is therefore not expected to have similar functionality as viral genome generated and virus-incorporated TF. In the absence of detailed structural and functional data on 6K and TF, it is at this point impossible to comment on the possibly separate roles played by these components in the life cycle of alphaviruses. Our work highlights the biophysical characteristics and functionalities of CHIKV 6K that are analogous to those displayed by viroporins and attempts to fundamentally characterize its cellular localization and multifaceted membrane interacting abilities. We hope that given the requirement for 6K for the propagation of the important human pathogen CHIKV, efforts will be made to utilize 6K as a drug target to develop therapeutic strategies in the future.

## Materials and methods

### Sequence analysis

Hydrophobicity plots corresponding to CHIKV 6K sequence, was generated using the ExPASy tool ProtScale (https://web.expasy.org/protscale/), and probable transmembrane domains were identified using DAS [47], TMHMM [48], TOPCONS [49] and PHOBIUS [50].

### DNA constructs

The cDNA corresponding to CHIKV 6K was obtained from a mammalian expression cassette encoding all structural proteins corresponding to CHIKV strain 37997 (CMV/R CHIKVC-E3-E2-6K-E1) [51]. This cassette was a kind gift from Dr. John Mascola, NIAID (National Institute of Allergy and Infectious Diseases, USA). The cDNA corresponding to 6K was subcloned into the BamHI and XhoI sites of the bacterial expression vector pGEX-6P2 (GE Healthcare), thus generating a construct with an N-terminal GST-tag. For mammalian expression, 6K cDNA was subcloned into the NheI and HindIII sites of pEGFP-N1 (Clontech), resulting in the insertion of an N-terminal EGFP tag. CHIKV glycoproteins E1 and E2 were subcloned into the BamHI/XbaI and EcoRI/XbaI sites, respectively, of pCDNA 3.1(+) (Invitrogen) with N-terminal myc-tag. All constructs were confirmed by sequencing.

### Expression and purification of GST-6K

Expression of GST-6K was induced in E. coli Rosetta pLysS cells. Cells were grown at 37°C until OD reached 0.8, when the temperature was reduced to 18°C, followed by addition of 1 mM IPTG. After 3 hours of induction, cells were pelleted and the expression of GST-6K was confirmed by western blotting, using an anti-GST antibody (Abcam, USA). The pelleted cells were resuspended in a lysis buffer containing 50 mM Tris pH 7.5, 100 mM NaCl, 1 mM DTT, 10% Glycerol and 1% CHAPS, lysed by sonication, followed by centrifugation to remove cellular debris, and a two-step purification process. In the first step, the soluble fraction was incubated with 200 μl of GST beads (Pierce Glutathione Agarose, Thermo Scientific) for 60 minutes at 4°C, with shaking, to pull down the fusion protein. The bound protein was eluted from beads using 10–20 mM glutathione in lysis buffer. The second step of purification involved size-exclusion chromatography on a Superdex-200 10/300 G/L column, using an ÄKTApurifier 10 (GE Healthcare). A buffer consisting of 50 mM Tris, pH 7.5 and 100 mM NaCl, at a flow rate of 0.5 ml/min was used for SEC elution.

### Electrophysiology experiments

Purified GST-6K was incorporated in the bilayer membrane as described previously [52]. The apparatus for electrophysiology experiments consisted of a polystyrene cuvette (Warner Instruments) with a thin wall separating two aqueous compartments. The polystyrene divider had a circular aperture with a diameter of 150μm. Both compartments were filled with 1 ml buffer containing 10 mM HEPES, pH 7.4, 500 mM KCl and 5 mM MgCl_2_ and connected to an integrating patch amplifier (Axopatch 200B, Axon Instruments) through a matched pair of Ag/AgCl electrodes. The cis chamber was connected to the head stage (CV-203BU) of the amplifier, while the trans-chamber was held at virtual ground. A solution of DPhPC (Avanti Polar Lipids, USA) and cholesterol (6:1) in n-decane (10 μl) was painted over the aperture to form the membrane. Reconstitution of GST-6K in BLM (Bilayer Membrane) was initiated by adding desired concentration (0.2, 0.85, 1.2, 2.6 ng/μl) of protein in BLM buffer, followed by mixing using a magnetic stirrer. A sudden shift in the membrane current indicated the incorporation of the channel in BLM. For amantadine inhibition experiments, GST-6K was added to a final concentration of 2.6 ng/μl. Amantadine to a final concentration of 1 μM was added to the BLM buffer containing GST-6K only after ensuring that the protein has properly integrated in the membrane. Channel current was recorded using Digidata (1440A, Axon Instruments), Low pass Bessel filter of 2 KHz and the acquisition software CLAMPEX (PCLAMP 10.2, Axon Instruments). The channel current was recorded at fixed applied membrane potentials in the range of -100 to +100mV at a sampling frequency of 10 KHz (temperature between 24–25°C). Open and closed states of the channel (s) were identified as described [52]. Data were analyzed using the software CLAMPFIT (PCLAMP 10.2, Axon Instruments), Origin 5.0 (Originlab Corp. USA) and Matlab.

### Liposome disruption assay

Generation of liposomes mimicking the membrane composition of the endoplasmic reticulum (ER) and plasma membrane, and liposome disruption assays, was carried out as described [31, 32]. All lipids were procured from Avanti Polar Lipids (Alabaster, AL, USA). For the assay, GST-6K, at a concentration of 0.1 μM to 10 μM, was incubated in the presence of dye-encapsulated liposomes for 30 minutes at 25°C, and end-point fluorescence was monitored at 585 nm. Purified GST was used as a control in all experiments. Experiments were carried out in triplicates on a Perkin Elmer fluorescence spectrophotometer using a quartz cuvette.

### Dynamic light scattering

DLS analysis was carried out in a Malvern Zetasizer (Nano ZS90). Briefly, freshly generated ER mimicking liposomes were incubated for 30 minutes alone, or in presence of different concentrations of GST-6K, calcium chloride or amantadine. All experiments were repeated thrice.

### Confocal Microscopy

HEK 293T cells were cultured in 12-well plates in DMEM, supplemented with 5% FBS and 1% Pen-Strep (GIBCO). Transfection was carried out using Lipofectamine 2000 (Life Technologies, USA) according to the manufacturer’s instructions.

Fixing and staining of cells for confocal microscopy were carried out as described [32]. Images were captured on a confocal laser scanning microscope (Leica SP5 Confocal Laser Scanning Microscope) using a 60X oil-immersion objective. Localization of 6K to cellular organelles or with E1/E2 was estimated by calculating the Pearson’s correlation coefficient, which measures the linear correlation between fluorescent channels. Values greater than 0.5 were considered to indicate a high degree of colocalization between fluorescent signals. General processing of images was carried out with the software ImageJ (http://rsb.info.nih.gov/ij/). All experiments were done in triplicates.

### CHIKV VLP production and purification

For production of VLPs, ~34.2 x 10^6^ adherent HEK 293T cells, maintained as above, were transfected with 32 μg purified plasmid DNA (CMV/R CHIKVC-E3-E2-6K-E1) mixed with 60 μl lipofectamine 2000, and incubated for 72 hours at 37°C, in presence of 5% CO_2_. Post incubation, media was harvested and subjected to cushioning on 15% sucrose at 1,00,000x g for 2 hours at 4°C. The resultant pellet was resuspended in a buffer consisting of 20 mM Tris, pH 7.5 and 100 mM NaCl.

### Electron microscopy

For negative staining, 4 μl of GST-6K (0.5 μM), mixed with ER liposomes and incubated for 25 minutes, was applied onto glow discharged, 400-mesh carbon-coated copper grids (Agar Scientific Ltd., UK). The sample was incubated for 4 minutes, and the excess drained off on a Whatman filter paper. 3 μl of 2% uranyl acetate was added for 1 minute, followed by washes (3X) with water, and the grid was air-dried for 1 minute. Micrographs were captured at a magnification of 29000x.

For cryo-freezing of CHIKV VLPs, 4.5 μl of purified VLPs (sucrose cushioned@100,000xg), at a concentration of 0.3mg/ml (absorbance at 280 nm), was applied to glow-discharged Quantifoil R2/2 holey carbon grids. Vitrification was carried out using a Vitrobot Mark IV (FEI/Thermofisher), with a blot time of 2.5s, by plunging grids into liquid ethane cooled by a surrounding bath of liquid nitrogen. Grids were transferred to a cryo-holder (Gatan model 626) and visualized on a FEI Tecnai F20 G^2^ FEG Transmission Electron Microscope, operated at 200 kV. Digital images were recorded on a CCD camera using TIA software under low-dose conditions at a magnification of 50,000x, with a defocus range of -3 to -5 μm.

### Reference-free 2D classification

2D classification of GST-6K was carried out using EMAN 2.2 [53]. Briefly, particles were boxed and extracted from micrographs. A total of 746 particles were selected for generating the 2D classes. All micrographs were CTF corrected and reference-free 2D classification was carried out using standard methods. A total of 6 iterations were done and 16 representative classes were chosen.

### Infection assays

Vero cells (African green monkey kidney epithelial cells) and Chikungunya virus (CHIKV, S 27 strain, accession no. AF369024.2) were kindly gifted by Dr. M. M. Parida, DRDE, Gwalior, India. Cells were maintained in Dulbecco’s modified Eagle’s medium (DMEM; PAN Biotech, Germany) supplemented with 5% Fetal bovine serum (FBS; PAN Biotech, USA), Gentamycin, and Penicillin-Streptomycin (Sigma, USA). Amantadine was purchased from Sigma Aldrich (now MERCK).

### Cellular cytotoxicity determination

Vero cells were seeded in 96 well plates (Corning, USA) and after the cells had reached 90% confluence, they were treated with different concentrations of amantadine for 24 hours and incubated at 37°C in 5% CO_2_. Cellular cytotoxicity assay was performed according to the protocol described earlier [54]. Cellular cytotoxicity was determined in triplicate and each experiment was repeated three times independently.

### CHIKV infection and Amantadine treatment

Vero cells at 90% confluence were grown in 24 well plates in complete DMEM (Pan Biotech, USA). For infection, cells were first washed two times with 1X PBS (Himedia, USA) and thereafter infected with CHIKV, at a Multiplicity of Infection (M.O.I) of 0.1, in serum-free medium. The infected cells were incubated at 37°C in 5% CO_2_ for 90 minutes with shaking at every 10 minutes interval. After 90 minutes, cells were washed twice with 1X PBS, and incubated with complete DMEM containing different concentrations of amantadine (2.5 μM to 200 μM). Thereafter, cell culture supernatants from CHIKV infected, drug-treated or untreated cells, were harvested at different hpi according to the experiment for viral titer estimation.

### Plaque assay

Plaque assay was performed according to the procedure mentioned before [55]. Briefly, vero cells seeded onto 6 well plates were infected with different dilutions of the harvested cell culture supernatants mentioned above. After infection, cells were washed twice with 1X PBS and overlaid with semi-solid media (DMEM containing 10% FBS and 2% methylcellulose). The cells were fixed once plaques were visible and countable (4 to 5 days post-infection). For fixing, the cells were first treated with 8% formaldehyde, followed by staining with 8% Crystal violet solution.

### qRT-PCR

Equal volume of samples (Mock, CHIKV infected, and infected as well as amantadine treated) was taken for viral RNA isolation using the QIAamp viral RNA isolation kit (Qiagen, USA) as per the manufacturer’s instructions. RT reaction was performed using the First Strand cDNA synthesis kit (Fermentas, USA) as per the manufacturer instructions. An equal volume of cDNA was used during qRT-PCR for amplifying E1 gene of CHIKV [56].

### Molecular dynamics simulations

In the absence of any experimentally determined three-dimensional structure for CHIKV 6K, two different structures predicted by the server Bhageerath-H [57] were utilized for simulation studies. Both structures contain a central alpha-helical segment with two other short helical stretches at the N- and the C-termini. The predicted three-dimensional structures of CHIKV 6K were embedded within a pre-equilibrated 392 POPC lipid bilayer. Peptide molecule(s) were placed perpendicular to the plane of the membrane. The entire system was solvated in water and 100 mM NaCl was added. After equilibration for 100 ns, the system was simulated without position restraints for 700 ns. After 700 ns, an electric field corresponding to 90–110 mV was switched on along the z-axis for facilitating ion conduction through the channel. All simulations were carried out for 1.5 μs, using the GROMACS package v5.1.1 [58]. Specific simulation steps followed has been outlined earlier [31, 59]. Gromacs analysis tools, UCSF Chimera [60] and VMD (Visual Molecular Dynamics) [61] were utilized for data analysis and molecular visualization.

## Supporting information

S1 Table Ion channel conductance at different concentrations of GST-6K on BLM at ±100mV. (TIF)

S1 Fig **Crosslinking of GST (left panel) and GST-6K (right panel) with 0.01% glutaraldehyde for 5 mins. Samples were resolved on a 12% SDS-PAGE. (A) Lanes 2 & 3 represent GST with and without crosslinking, (B) Lanes 2 & 3 represent GST-6K with and without crosslinking**.
(TIF)

S2 Fig Representative current vs. time traces obtained from bilayer electrophysiology of GST at various concentrations (0, 0.2, 0.85, 1.2, 2.6 ng/μl), and at applied membrane potentials of -100 mV and +100 mV. (TIF)

S3 Fig DSSP analysis of 6K hexamer in membrane showing the evolution of the protein secondary structure during the simulation time period. (TIF)

## References

[1] Hua C & Combe B. (2017) Chikungunya Virus-Associated Disease. Current Rheumatology Reports. 10.1007/s11926-017-0694-0 . 28983760

[2] Figueiredo LTM. (2017) Large outbreaks of chikungunya virus in Brazil reveal uncommon clinical features and fatalities. Revista da Sociedade Brasileira de Medicina Tropical. 10.1590/0037-8682-0397-2017 . 29160502

[3] De Brito CAA. (2017) Alert: Severe cases and deaths associated with Chikungunya in Brazil. Revista da Sociedade Brasileira de Medicina Tropical. 10.1590/0037-8682-0479-2016 29160503

[4] Kaur N, Jain J, Kumar A, Narang M, Zakaria MK, Marcello A, Kumar D, Gaind R, Sunil S. (2017) Chikungunya outbreak in Delhi, India, 2016: report on coinfection status and comorbid conditions in patients. New Microbes and New Infections. 10.1016/j.nmni.2017.07.007 . 29158907 PMC5682881

[5] Jose J, Snyder JE, Kuhn RJ. (2009) A structural and functional perspective of alphavirus replication and assembly. Future Microbiology. 10.2217/fmb.09.59 . 19722838 PMC2762864

[6] Firth AE, Chung BYW, Fleeton MN, Atkins JF. (2008) Discovery of frameshifting in Alphavirus 6K resolves a 20-year enigma. Virology Journal. 10.1186/1743-422X-5-108 . 18822126 PMC2569925

[7] Snyder JE, Kulcsar KA, Schultz KLW, Riley CP, Neary JT, Marr S, Jose J, Griffin DE, Kuhn RJ. (2013) Functional Characterization of the Alphavirus TF Protein. Journal of Virology. 10.1128/JVI.00449-13 . 23720714 PMC3719798

[8] Sun S, Xiang Y, Akahata W, Holdaway H, Pal P, Zhang X, Diamond MS, Nabel GJ, Rossmann MG. (2013) Structural analyses at pseudo atomic resolution of Chikungunya virus and antibodies show mechanisms of neutralization. Elife. 10.7554/eLife.00435 . 23577234 PMC3614025

[9] Taylor A, Liu X, Zaid A, Goh LYH, Hobson-Peters J, Hall RA, Merits A, Mahalingam S. (2017). Mutation of the n-terminal region of chikungunya virus capsid protein: Implications for vaccine design. MBio. 10.1128/mBio.01970-16 . 28223458 PMC5358915

[10] Sharma R, Fatma B, Saha A, Bajpai S, Sistla S, Dash PK, Parida M, Kumar P, Tomar S. (2016) Inhibition of chikungunya virus by picolinate that targets viral capsid protein. Virology. 10.1016/j.virol.2016.08.029 . 27614702

[11] Zheng Y & Kielian M. (2015) An alphavirus temperature-sensitive capsid mutant reveals stages of nucleocapsid assembly. Virology. 10.1016/j.virol.2015.05.011 . 26051211 PMC4567448

[12] Hikke MC, Geertsema C, Wu V, Metz SW, van Lent JW, Vlak JM, Pijlman GP. (2016) Alphavirus capsid proteins self-assemble into core-like particles in insect cells: A promising platform for nanoparticle vaccine development. Biotechnology Journal. 10.1002/biot.201500147 26287127

[13] Dudha N, Rana J, Rajasekharan S, Gabrani R, Gupta A, Chaudhary VK, Gupta S. (2015) Host–pathogen interactome analysis of Chikungunya virus envelope proteins E1 and E2. Virus Genes. 10.1007/s11262-014-1161-x . 25563600

[14] Agarwal A, Sharma AK, Sukumaran D, Parida M, Dash PK. (2016) Two novel epistatic mutations (E1:K211E and E2:V264A) in structural proteins of Chikungunya virus enhance fitness in Aedes aegypti. Virology. 10.1016/j.virol.2016.06.025 . 27423270

[15] Lorente E, Barriga A, García-Arriaza J, Lemonnier FA, Esteban M, López D. (2017) Complex antigen presentation pathway for an HLA-A*0201-restricted epitope from Chikungunya 6K protein. PLOS Neglected Tropical Diseases. 10.1371/journal.pntd.0006036 . 29084215 PMC5679651

[16] Melton JV., Ewart GD, Weir RC, Board PG, Lee E, Gage PW. (2002) Alphavirus 6K proteins form ion channels. Journal of Biological Chemistry. 10.1074/jbc.M20784720 12228229

[17] Yao JS, Strauss EG, Strauss JH. (1996) Interactions between PE2, E1, and 6K required for assembly of alphaviruses studied with chimeric viruses. Journal of Virology. Available: http://www.pubmedcentral.nih.gov/articlerender.fcgi?artid=190863&tool=pmcentrez&rendertype=abstract. . 8892914 10.1128/jvi.70.11.7910-7920.1996 PMC190863

[18] Sanz MA, Carrasco L. (2001) Sindbis Virus Variant with a Deletion in the 6K Gene Shows Defects in Glycoprotein Processing and Trafficking: Lack of Complementation by a Wild-Type 6K Gene in trans. Journal of Virology. 10.1128/JVI.75.16.7778 . 11462055 PMC115018

[19] Liljeström P, Lusa S, Huylebroeck D, Garoff H. (1991) In vitro mutagenesis of a full-length cDNA clone of Semliki Forest virus: the small 6,000-molecular-weight membrane protein modulates virus release. Journal of Virology. 65: 4107–4113. . 2072446 10.1128/jvi.65.8.4107-4113.1991 PMC248843

[20] Ivanova L, Lustig S, Schlesinger MJ. (1995) A Pseudo-Revertant of a Sindbis Virus 6K Protein Mutant, Which Corrects for Aberrant Particle Formation, Contains Two New Mutations That Map to the Ectodomain of the E2 Glycoprotein. Virology. 10.1006/viro.1995.1025 . 7856077

[21] Gaedigk-Nitschko K, Schlesinger MJ. (1991) Site-directed mutations in sindbis virus E2 glycoprotein cytoplasmic domain and the 6K protein lead to similar defects in virus assembly and budding. Virology. 10.1016/0042-6822(91)90133-V . 1647069

[22] Taylor A, Melton JV., Herrero LJ, Thaa B, Karo-Astover L, Gage PW, Nelson MA, Sheng KC, Lidbury BA, Ewart GD, McInerney GM, Merits A, Mahalingam S. (2016) Effects of an In-Frame Deletion of the 6k Gene Locus from the Genome of Ross River Virus. Journal of Virology. 10.1128/JVI.03192-15 . 26865723 PMC4810561

[23] Guo TC, Johansson DX, Haugland Ø, Liljeström P, Evensen Ø. (2014) A 6K-deletion variant of salmonid alphavirus is non-viable but can be rescued through RNA recombination. PLoS One. 10.1371/journal.pone.0100184 . 25009976 PMC4091863

[24] Antoine AF, Montpellier C, Cailliau K, Browaeys-Poly E, Vilain JP, Dubuisson J. (2007) The Alphavirus 6K protein activates endogenous ionic conductances when expressed in Xenopus oocytes. Journal of Membrane Biology. 10.1007/s00232-007-9003-6 . 17483865

[25] Sanz MA, Madan V, Carrasco L, Nieva JL. (2003) Interfacial domains in sindbis virus 6K protein: Detection and functional characterization. Journal of Biological Chemistry. 10.1074/jbc.M206611200 . 12424249

[26] Ramsey J, Renzi EC, Arnold RJ, Trinidad JC, Mukhopadhyay S. (2011) Palmitoylation of Sindbis Virus TF Protein Regulates Its Plasma Membrane Localization and Subsequent Incorporation into Virions. J. Virol. 2017, 91, e02000 10.1371/journal.pntd.0006036 PMC5244351 27852864

[27] Wang K, Xie S & Sun B. Viral proteins function as ion channels. Biochimica et Biophysica Acta (BBA)—Biomembranes. 10.1016/j.bbamem.2017.11.009 . 20478263 PMC7094589

[28] Jing X, Ma C, Ohigashi Y, Oliveira FA, Jardetzky TS, Pinto LH, Lamb RA. (2008) Functional studies indicate amantadine binds to the pore of the influenza A virus M2 proton-selective ion channel. Proceedings of the National Academy of Sciences. 10.1073/pnas.0804958105 . 18669647 PMC2492755

[29] Rochet J. C., Brownie E. R., Oikawa K., Hicks L. D., Fraser M. E., James M. N., Kay C. M., Bridger W. A., and Wolodko W. T. (2000) Pig heart CoA transferase exists as two oligomeric forms separated by a large kinetic barrier. *Biochemistry* 39, 11291–11302. 10.1021/bi0003184 10985774

[30] Griffin SD, Beales LP, Clarke DS, Worsfold O, Evans SD, Jaeger J, Harris MP, Rowlands DJ. (2003) The p7 protein of hepatitis C virus forms an ion channel that is blocked by the antiviral drug, Amantadine. FEBS Lett. 30;535(1–3):34–8. 10.1016/s0014-5793(02)03851-6 12560074

[31] Shukla A, Padhi AK, Gomes J, Banerjee M. (2014) The VP4 Peptide of Hepatitis A Virus Ruptures Membranes through Formation of Discrete Pores. Journal of Virology. 10.1128/JVI.01896-14 . 25122794 PMC4248947

[32] Shukla A, Dey D, Banerjee K, Nain A, Banerjee M. (2015) The C-terminal region of the non-structural protein 2B from Hepatitis A Virus demonstrates lipid-specific viroporin-like activity. Scientific Reports. 5: 15884 10.1038/srep15884 . 26515753 PMC4626808

[33] Brown RS, Wan JJ, Kielian M. (2018) The Alphavirus Exit Pathway: What We Know and What We Wish We Knew. Viruses. 10, 89 10.3390/v10020089 . 29470397 PMC5850396

[34] Lobigs M, Hongxing Z, Garoff H. (1990) Function of Semliki Forest Virus E3 Peptide in Virus Assembly: Replacement of E3 with an Artificial Signal Peptide Abolishes Spike Heterodimerization and Surface Expression of El. Journal of Virology, p. 4346–4355. . 2200886 10.1128/jvi.64.9.4346-4355.1990 PMC247902

[35] Fields W, Kielian M. (2013) A Key Interaction between the Alphavirus Envelope Proteins Responsible for Initial Dimer Dissociation during Fusion. Journal of Virology p. 3774–3781. 10.1128/JVI.03310-12 . 23325694 PMC3624238

[36] Pellegrini-Calace M, Maiwald T, Thornton JM. (2009) Pore-Walker: a novel tool for the identification and characterization of transmembrane protein channels from their three-dimensional structure&rdquo. PLOS Computational. Biology. 10.1371/journal.pcbi.1000440 . 19609355 PMC2704872

[37] Tarahovsky YS, Yagolnik EA, Muzafarov EN, Abdrasilov BS, Kim YA (2012). Calcium-dependent aggregation and fusion of phosphatidylcholine liposomes induced by complexes of flavonoids with divalent iron. Biochim Biophys Acta—Biomembranes. 10.1016/j.bbamem.2011.12.001 . 22179037

[38] Bron R, Kendal AP, Klenk HD, Wilschut J. (1993) Role of the m2 protein in influenza virus membrane fusion: Effects of amantadine and monensin on fusion kinetics. Virology. 10.1006/viro.1993.1435 . 8337846

[39] Más V. Melero JA. (2013) Entry of Enveloped Viruses into Host Cells: membrane Fusion. Subcellular Biochemistry. 10.1007/978-94-007-6552-8_16 . 23737062 PMC7121288

[40] Kumar CS, Dey D, Ghosh S, Banerjee M. (2017) Breach: Host Membrane Penetration and Entry by Nonenveloped Viruses. Trends in Microbiology. 10.1016/j.tim.2017.09.010 . 29079499

[41] Nieto-Torres JL, Verdiá-Báguena C, Castaño-Rodriguez C, Aguilella VM, Enjuanes L. (2015) Relevance of viroporin ion channel activity on viral replication and pathogenesis. Viruses. 10.3390/v7072786 . 26151305 PMC4517115

[42] Dey D, Banerjee M. (2016) Inhibitor-Based Therapeutics for Treatment of Viral Hepatitis. Journal of Clinical and Translational Hepatology. 10.14218/JCTH.2016.00025 . 27777893 PMC5075008

[43] Vigant F, Santos NC, Lee B. (2015) Broad-spectrum antivirals against viral fusion. Nature Reviews Microbiology. pp. 426–437. 10.1038/nrmicro3475 . 26075364 PMC4554337

[44] Thomaston J.L., Polizzi N.F., Konstantinidi A., Wang J., Kolocouris A., DeGrado W.F. Inhibitors of the M2 Proton Channel Engage and Disrupt Transmembrane Networks of Hydrogen-Bonded Waters. (2018) J. Am. Chem. Soc. 140: 15219–15226. 10.1021/jacs.8b06741 30165017 PMC6497056

[45] Ciampor F, Bayley PM, Nermut M V., Hirst EMA, Sugrue RJ, Hay AJ. (1992) Evidence that the amantadine-induced, M2-mediated conversion of influenza A virus hemagglutinin to the low pH conformation occurs in an acidic trans golgi compartment. Virology. 10.1016/0042-6822(92)90730-D . 1566569

[46] Xue ST, He WY, Ma LL, Wang HQ, Wang B, Zheng BH, Ji XY, Zhang T, Li YH, Jiang JD, Li ZR. (2013) Synthesis and Anti-Influenza Virus Activities of a Novel Class of Gastrodin Derivatives. 10.3390/molecules18043789 . 23531598 PMC6270409

[47] Cserzo M, Wallin E, Simon I, von Heijne G, Elofsson A. (1997) Prediction of transmembrane alpha-helices in prokaryotic membrane proteins: the dense alignment surface method. Protein Engineering Design and Selection. 10.1093/protein/10.6.673 . 9278280

[48] Krogh A, Larsson B, Heijne GV, Sonnhammer EL. (2001) Predicting transmembrane protein topology with a hidden Markov model: application to complete genomes. Journal of Molecular Biology. 10.1006/jmbi.2000.4315 . 11152613

[49] Tsirigos KD, Peters C, Shu N, Käll L, Elofsson A. (2015) The TOPCONS web server for consensus prediction of membrane protein topology and signal peptides. Nucleic Acids Research (2015). 10.1093/nar/gkv485 . 25969446 PMC4489233

[50] Käll L, Krogh A, Sonnhammer ELL. (2007) Advantages of combined transmembrane topology and signal peptide prediction-the Phobius web server. Nucleic Acids Research. 10.1093/nar/gkm256 . 17483518 PMC1933244

[51] Akahata W, Yang ZY, Andersen H, Sun S, Holdaway HA, Kong WP, Lewis MG, Higgs S, Rossmann MG, Rao S, Nabel GJ. (2010) A virus-like particle vaccine for epidemic Chikungunya virus protects nonhuman primates against infection. Nature Medicine. 10.1038/nm.2105.17 . 20111039 PMC2834826

[52] Malik C, Ghosh S. (2013) S6 Peptide derived from KvAP Channel forms Multi-Channels on Bilayer Lipid Membrane and shows Cooperativity in Gating. PLoS One. 8: e78845 10.1371/journal.pone.0078845 . 24265723 PMC3827124

[53] Tang G, Peng L, Baldwin PR, Mann DS, Jiang W, Rees I, Ludtke SJ. (2007) EMAN2: an extensible image processing suite for electron microscopy. J Struct Biol. 10.1016/j.jsb.2006.05.009 . 16859925

[54] Das I, Basantray I, Mamidi P, Nayak TK, Pratheek BM, Chattopadhyay S, Chattopadhyay S. (2014) Heat shock protein 90 positively regulates Chikungunya virus replication by stabilizing viral non-structural protein nsP2 during infection. PLoS One. 10.1371/journal.pone.0100531 . 24959709 PMC4069056

[55] Chattopadhyay S, Weller SK. (2006) DNA binding activity of the herpes simplex virus type 1 origin binding protein, UL9, can be modulated by sequences in the N terminus: correlation between transdominance and DNA binding. Journal of Virology. 10.1128/JVI.80.9.4491-4500.2006 . 16611909 PMC1471996

[56] Mishra P, Kumar A, Mamidi P, Kumar S, Basantray I, Saswat T, Das I, Nayak TK, Chattopadhyay S, Subudhi BB, Chattopadhyay S. Inhibition of Chikungunya Virus Replication by 1-[(2 Methylbenzimidazol-1-yl) Methyl]-2-Oxo-Indolin-3-ylidene] Amino] Thiourea (MBZM-N-IBT). *Sci. Rep*. 6, 20122; 10.1038/srep20122 (2016). 26843462 PMC4740769

[57] Jayaram B, Dhingra P, Mishra A, Kaushik R, Mukherjee G, Singh A, Shekhar S. (2014) Bhageerath-H: a homology/ab initio hybrid server for predicting tertiary structures of monomeric soluble proteins. BMC Bioinformatics. 10.1186/1471-2105-15-S16-S7 25521245 PMC4290660

[58] Abraham MJ, Murtola T, Schulz R, Páll S, Smith JC, Hess B, Lindahl E. (2015) Gromacs: High performance molecular simulations through multi-level parallelism from laptops to supercomputers. SoftwareX. 10.1016/j.softx.2015.06.001

[59] Bajaj S, Dey D, Bhukar R, Kumar M, Banerjee M. (2016) Non-Enveloped Virus Entry: Structural Determinants and Mechanism of Functioning of a Viral Lytic Peptide. Journal of Molecular Biology. 10.1016/j.jmb.2016.06.006 . 27320388

[60] Pettersen EF, Goddard TD, Huang CC, Couch GS, Greenblatt DM, Meng EC, Ferrin TE. (2004) UCSF Chimera—A Visualization System for Exploratory Research and Analysis. Journal of Computational Chemistry. 10.1002/jcc.20084 . 15264254

[61] Humphrey W, Dalke A, Schulten K. (1996) VMD: Visual molecular dynamics. Journal of Molecular Graphics. 10.1016/0263-7855(96)00018-5 . 8744570

