# Supplementary figures and images for: The effect of amantadine on an ion channel protein from Chikungunya virus
Source: PLoS Negl Trop Dis. 2019 Jul 24;13(7):e0007548. doi: 10.1371/journal.pntd.0007548 (PMC6655611; doi:10.1371/journal.pntd.0007548)

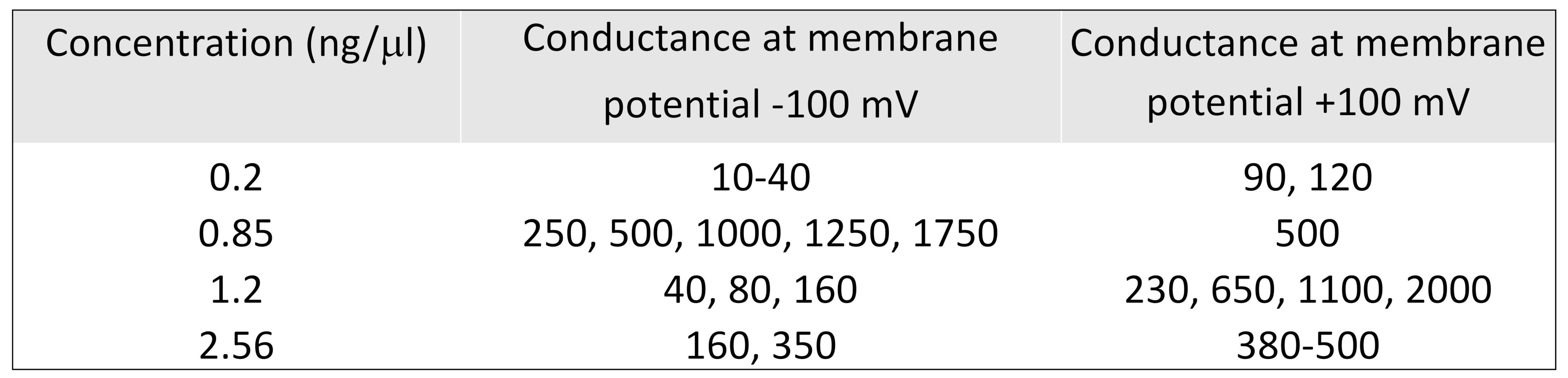

Supplement: S1 Table — (TIF) [file pntd.0007548.s001.tif]

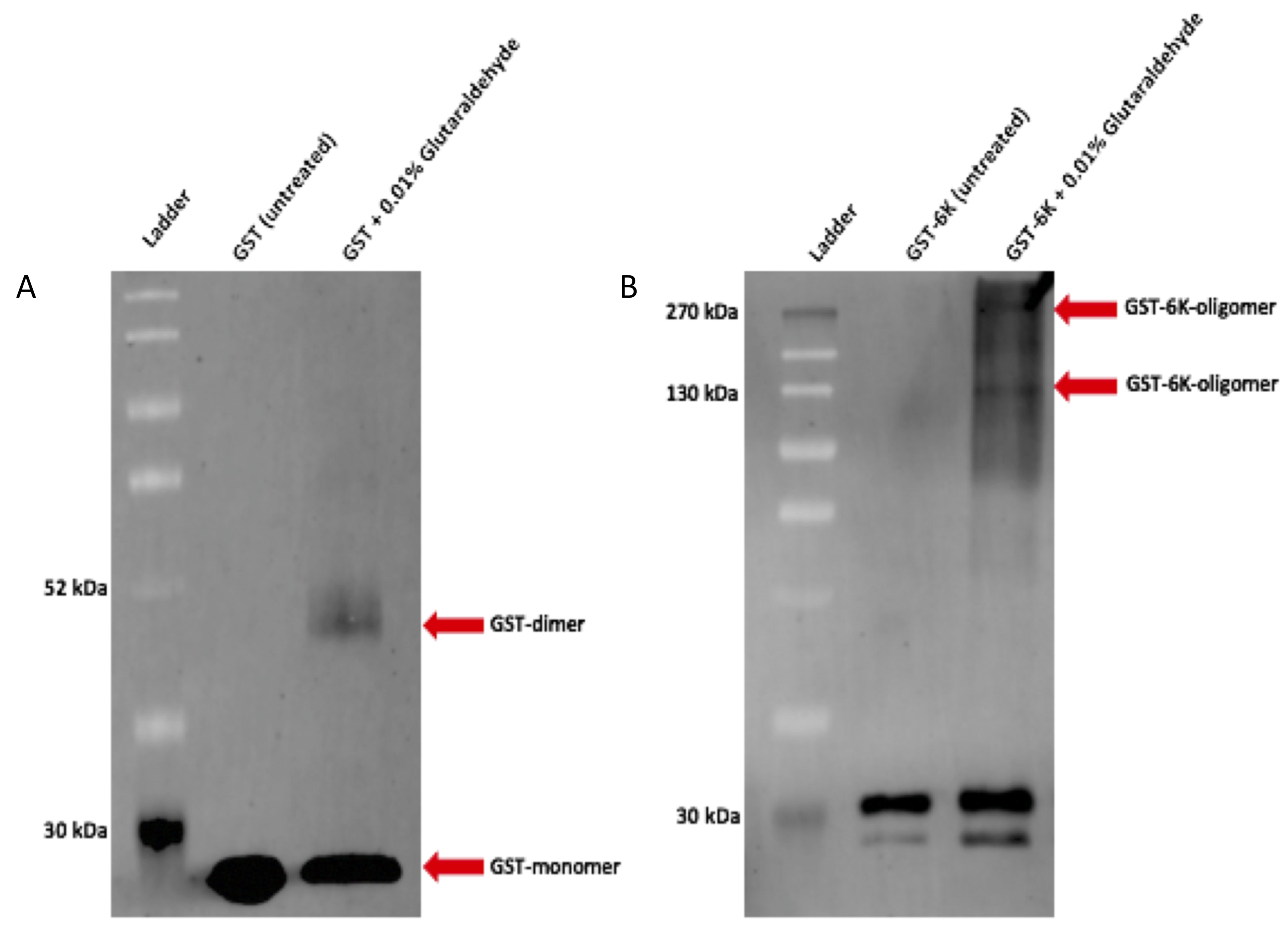

Supplement: S1 Fig — Crosslinking of GST (left panel) and GST-6K (right panel) with 0.01% glutaraldehyde for 5 mins. Samples were resolved on a 12% SDS-PAGE. (A) Lanes 2 & 3 represent GST with and without crosslinking, (B) Lanes 2 & 3 represent GST-6K with and without crosslinking. (TIF) [file pntd.0007548.s002.tif]

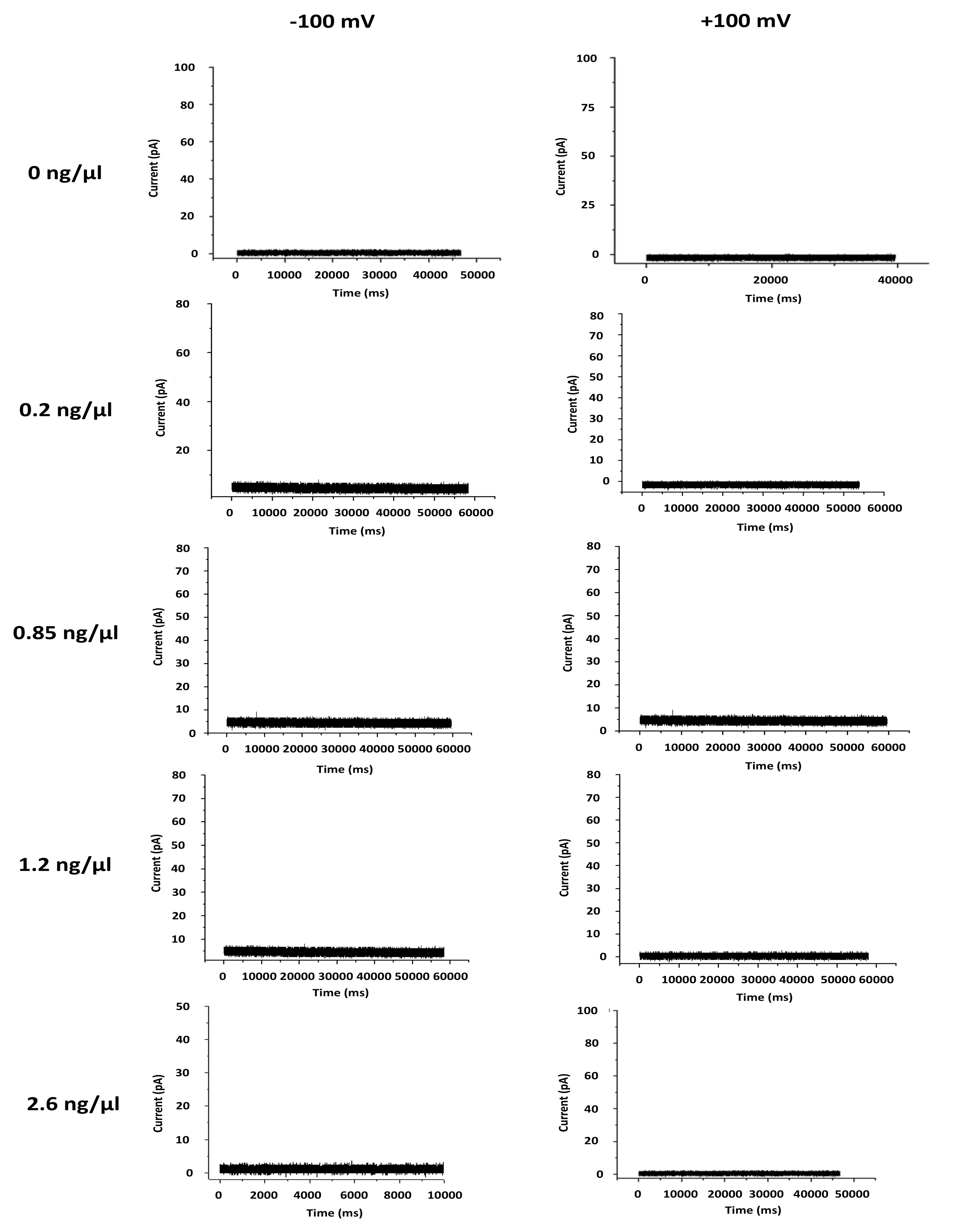

Supplement: S2 Fig — (TIF) [file pntd.0007548.s003.tif]

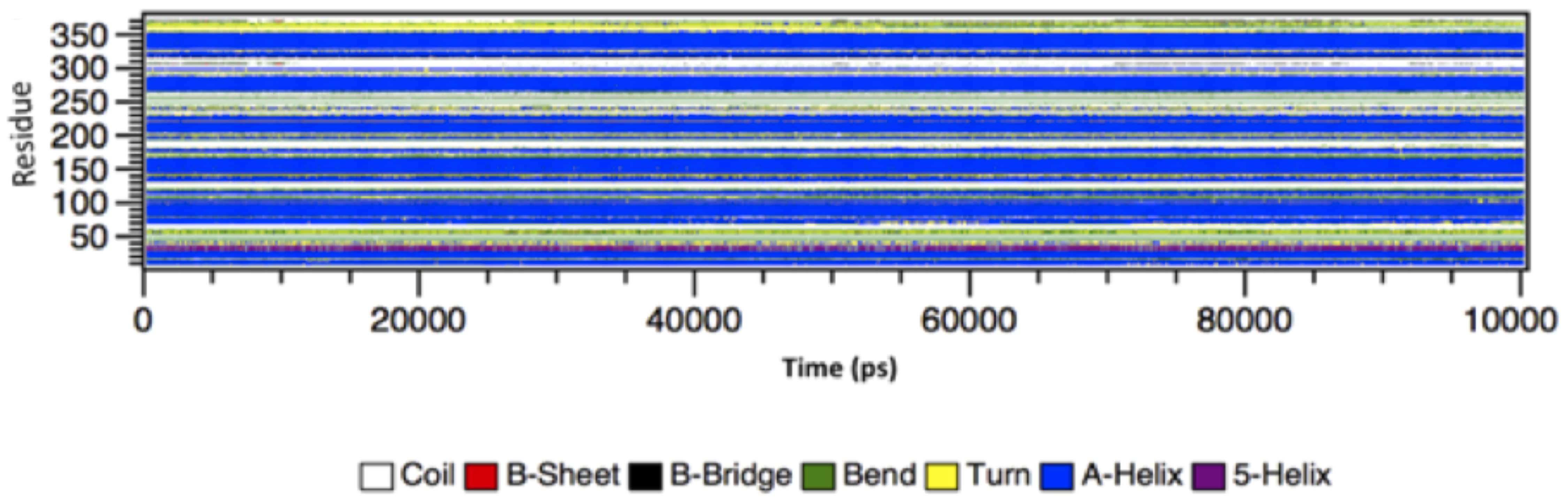

Supplement: S3 Fig — (TIF) [file pntd.0007548.s004.tif]
